# Synthesis of MnO/C/Co_3_O_4_ nanocomposites by a Mn^2+^-oxidizing bacterium as a biotemplate for lithium-ion batteries

**DOI:** 10.1080/14686996.2021.1927175

**Published:** 2021-06-04

**Authors:** Jin Liu, Tong Gu, Xiaowen Sun, Li Li, Fan Xiao, Zhiyong Wang, Lin Li

**Affiliations:** State Key Laboratory of Agricultural Microbiology, Huazhong Agricultural University, Wuhan, China

**Keywords:** Biogenic manganese oxides, biotemplate, hollow porous nanostructure, metal ion incorporation, lithium-ion battery, 40 Optical, magnetic and electronic device materials, 102 Porous / Nanoporous / Nanostructured materials, 207 Fuel cells / Batteries / Super capacitors

## Abstract

The biotemplate and bioconversion strategy represents a sustainable and environmentally friendly approach to material manufacturing. In the current study, biogenic manganese oxide aggregates of the Mn^2+^-oxidizing bacterium *Pseudomonas* sp. T34 were used as a precursor to synthesize a biocomposite that incorporated Co (CMC-Co) under mild shake-flask conditions based on the biomineralization process of biogenic Mn oxides and the characteristics of metal ion subsidies. X-ray photoelectron spectroscopy, phase composition and fine structure analyses demonstrated that hollow MnO/C/Co_3_O_4_ multiphase composites were fabricated after high-temperature annealing of the biocomposites at 800°C. The cycling and rate performance of the prepared anode materials for lithium-ion batteries were compared. Due to the unique hollow structure and multiphasic state, the reversible discharge capacity of CMC-Co remained at 650 mAh g^–1^ after 50 cycles at a current density of 0.1 Ag^–1^, and the coulombic efficiency remained above 99% after the second cycle, indicating a good application potential as an anode material for lithium-ion batteries.

## Graphical abstract

**Figure:**
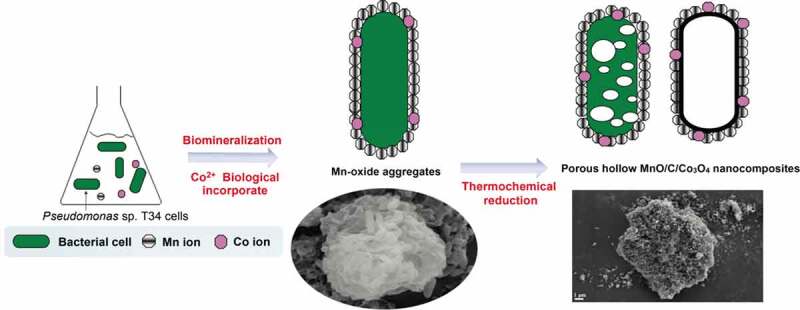

## 1. Introduction

Mn oxides are transition-metal oxides with rich reserves and a soil content second only to iron. The catalysis and oxidization of Mn^2+^ is primarily mediated by microorganisms and dominates the formation of Mn minerals in natural environments [1]. Previous studies of several Mn(II)-oxidizing bacteria have characterized the biogenic Mn(II) oxidation of soluble Mn(II) to Mn(IV) oxides as an enzymatically catalyzed biochemical process [2–4]. As the oxidation proceeds, Mn biooxides assemble on the cell surface, forming an Mn-oxide deposit layer. The layers on the cell surface become gradually superimposed, resulting in various microspherical aggregates. These bacterial spheroids with high-potential Mn oxides further serve as preliminary nucleation centers to oxidize and adsorb other metal ions or organic substrates, thereby successively forming bulkier aggregates [5]. The enzyme-mediated oxidation reactions all appear to be related to the cell surface and thus often occur in association with other mineral phases (e.g. Fe oxides) in desert varnishes, sediments, soil and ocean nodules, as mineral/rock coatings or in association with microorganisms [1,5–7]. Many previous investigations have verified that biogenic Mn oxides are produced by Mn^2+^-oxidizing bacteria as fine granular, film-adhesive, and dendritic aggregations or blocky and spherical complexes, the Mn biooxide was determined to be a poorly crystalline layered manganate with a high degree of c-axis stacking disorder and a moderate number of vacancies [5,8,9]. Generally, these biogenic minerals have a high reaction activity due to their strong oxidizing nature, large superficial area and high amount of negative charge that is capable of adsorbing various metal ions [7,9]. The Mn biooxide materials with different structures and morphologies have been used in various fields including catalysis, adsorption, sensors, and ion exchange [10–13]. Previous studies have verified the possibility that the oxidizing activity of microorganisms is utilized to prepare high-activity energy materials. For example, Zhou et al. used recombinant *Escherichia coli* as template to synthesize LiFeO_4_ nanostructures for lithium-ion electrode materials by adding Fe^3+^ [14]. In addition, a Mn oxidizing fungus was also used as biological oxidizer and template to prepare a special lithium-ion sieve with a microtube morphology by a solid-state transformation method [13]. Therefore, biogenic Mn oxides may also be used as promising materials for electrochemical energy storage [15].

Oxide based composites are attractive anode materials, which have been rapidly developed in recent years [16–18]. However, when biogenic Mn oxides are proposed to be anode electrodes, some possible shortcomings should be considered. These include the following: (1) the defect structure is metastable, and the cation-deficient structures usually only tolerate a limited temperature range (usually <100°C) without experiencing some degree of cation-vacancy loss; (2) the electrical conductivity decreases with an increase in vacancy content; and (3) the high defect and high activity of the surface layer could cause unstable electrochemical cycling performance [19]. Moreover, although MnO has a theoretically high specific capacity (756 mAh g^–1^) and a relatively low operation potential (1.032 V *vs* Li/Li^+^) compared with other transition metal oxides [20,21], it is also recognized for its shortcomings such as a low utility rate, poor rate capacity and short cyclic performance when used as an electrode of lithium-ion batteries [22–24]. Therefore, for the purpose of using biogenic Mn-oxides for electrochemical energy storage, these technical issues need to be addressed so that the high ion-exchange capacity from the cation vacancies of biogenic Mn-oxides can be used while avoiding the potential impairment of their electrochemical capacities.

An effective approach to solve these problems is to use the specific structure of certain organisms as a biotemplate to synthesize materials with a special morphology, followed by modification with other metal ions. The programmed growth and various life activities of naturally occurring organisms provide multidimensional materials with elaborate architectures from nano to micrometers. The superplasticity and creep properties of the nanoscale materials enable them to exhibit a strong resistance to volume change [25] as well as to improve the energy density, reduce the polarization phenomenon in the chemical reaction and enhance the electrode capability [26]. The second advantage of biotemplate strategy is constructing nanocomposites with a carbonaceous matrix, carbon-incorporation is an advanced approach to improve the electrochemical properties of electrode materials [27–29]. In addition to the structural specificity, the biotemplate-derived materials have other advantages including extensive template sources as well as a simple and cost-effective preparation processes. Therefore, these biotemplate materials can be used to realize the performance optimization of electrode materials, as demonstrated in a recent attempt using spirogyra as a template to prepare the MnO/C microtubes for lithium-ion batteries [30]. Moreover, the coexistence of Mn oxide with other metal oxides also significantly improved the specific discharge capacity, cycling stability and electrochemical performance of lithium storage [31–33]. Thus, the applications of biotemplate systems combining simultaneous metal-ion incorporation have appeal for improving the electrochemical properties of Mn oxide electrode materials.

**Scheme 1.**
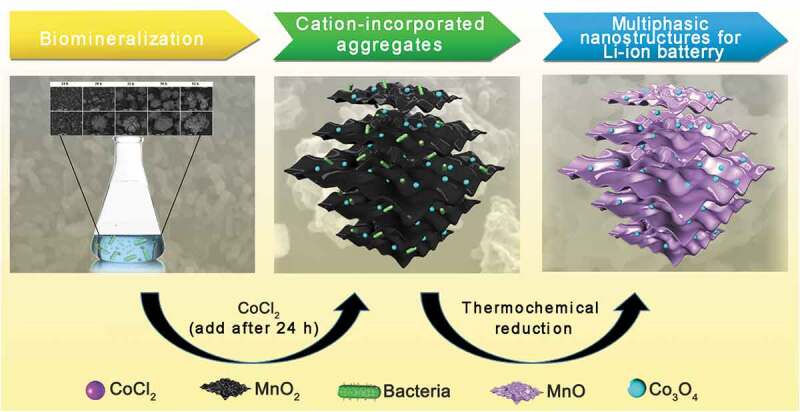
Schematic illustration of the procedures used in the process of MnO-based multiphasic CMC-Co fabrication

In this study, a biotemplating method was used to fabricate a hollow MnO/C/Co_3_O_4_ composite (Scheme 1) that was used as an anode material for lithium-ion batteries. A Mn^2+^-oxidizing bacterium *Pseudomonas* sp. T34 was characterized for its ability to form regular microspherical aggregates with diameters of 10 to 15 μm after cultivation under laboratory shake-flask culture conditions. The aggregate used in this work had three distinguishing features when it was used as biological template: (i) Easy availability. The Mn oxidizing effect could occur after cultivating the strain for 24 h, the aggregate could be obtained by centrifugal collection of precipitates after 48 h, they are easy to cultivate and harvest. (ii) The aggregate was assembled by Mn biooxide deposited on the cell surface [5], the decomposition of organic matter during high-temperature annealing could fabricate a hollow porous nanostructures; (iii) Special metal ion incorporation route. The presence of lattice vacancies in biogenic Mn oxides as well as the anionic groups of microorganisms in aggregate enhanced the metal-ion adsorption capacity [9,34,35]. Co^2+^ can be oxidized to Co^3+^ and introduced into Mn oxides by enzymatic reaction through a redox reaction, which indicates that the metal ions were not only loosely adsorbed on the biogenic Mn-oxide surface [36]. Hence, we believe that the synthesis of novel MnO/C/Co_3_O_4_ electrode materials derived from Mn^2+^-oxidizing aggregates will be a good example to achieve combination of nature and technology. In this study, following the biomineralization combined with simultaneous Co^2+^ incorporation, the aggregate precursor was carbonized in an Ar atmosphere at different high temperatures to fabricate hollow porous carbonaceous MnO-based Co_3_O_4_ multiphasic composites. The morphology and structure of the material formed by carbonization at 800°C were characterized by multiple assays using X-ray photoelectron spectroscopy (XPS), phase composition and fine structure analyses, among others. The prepared materials were further used as electrodes for lithium-ion batteries for the detection of their electrochemical capacities. The influence of the material structure on the electrochemical properties was investigated. The possible mechanism underlying the electrochemical capacity differentiation was also discussed.

## 2. Experimental details

### 2.1. Chemicals, bacterial strains, culture conditions and Mn^2+^-oxidizing activity assay

All chemicals were of analytical grade and were used as received without purification. CoCl_2_^.^6H_2_O (≥99%) was purchased from the Sigma–Aldrich Co. and was used as a Co source in the leptothrix medium for the cation-incorporated composite.

The wild-type strain *Pseudomonas* sp. T34, which is referred to as CCTCC (China Center for Type Culture Collection, Beijing, China) M2014168 that was originally isolated from a Fe/Mn nodule-surrounding brown soil sample, was used as the host strain for Mn-oxide aggregate production in laboratory shaker-flask trials. The strain was grown in *Leptothrix* medium supplemented with 1 mmol L^–1^ MnCl_2_ at 28°C and shaken at 150 rpm. A recipe for *Leptothrix* growth medium was provided by Capsi et al [3]. The Mn^2+^-oxidizing activity was measured over 7 days, while cells were grown in liquid *Leptothrix* medium in the presence of 1 mmol L^–1^ Mn^2+^ as the final concentration. A standard Leucoberbelin Blue spectrophotometry assay was performed for Mn-oxide quantification as described previously [37].

### 2.2. Preparation of the *MnO/C/Co_3_O_4_* composite

The preparation and use of the MnO/C/Co_3_O_4_ hollow porous composite is illustrated in Scheme 1. First, for the preparation of Mn oxide aggregates, the suspension cultures were harvested after cultivating *Pseudomonas* sp. T34 cells for 2 days. The resulting biogenic MnO_2_/T34 composites (‘BMC’ in brief) were rinsed three times with double distilled water and then dried at 80°C for 10 h. Approximately 0.15 g of biogenic MnO_2_/T34 composite could be obtained from a typical 1 L culture (the final concentration of Mn^2+^ is 1 mmol L^–1^). The biogenic MnO_2_/T34 cation-incorporated aggregates (BMC-Co) were prepared, except that additional CoCl_2_ was added to the culture after 24 h at a final concentration of 1 mmol L^–1^ during the incubation. Second, for the thermochemical process, a MnO/C composite was obtained by calcination of the BMC in a tube furnace at 800°C (temperatures of 400°C, 800°C and 1000°C were investigated, and the corresponding materials were defined as C400, C800 and C1000, respectively) for 4 h at 5°C min^–1^ under an Ar flow. The MnO/C/Co_3_O_4_ hollow porous composite (CMC-Co) was prepared by calcination of the BMC-Co in a tube furnace at 800°C. To produce 1 g CMC-Co, according to the method above, need 2.648 g BMC, 1.02 g Mn and 1.09 g Co ions. The total time required for CMC-Co preparation is 52 h.

### 2.3. Material characterization

The kinetics of the Mn^2+^-oxidizing activity were monitored by a UV/Vis spectrophotometer (DU-800 Nucleic Acids/Protein Analyzer, Beckman Coulter, America). The sample phase purity and properties were collected using X-ray powder diffraction (XRD, Bruker D8 Advance diffractometer with Cu Kα, λ = 0.15418 nm, Bruker, Germany). XPS analysis of the obtained materials was conducted with a VG Multilab2000 spectrometer (Thermo Electron Corporation, America) with an Al Kα X-ray source (1486 eV) and a base pressure of 3 × 10^−9^ Torr in the analytical chamber [38]. The charging effect was reduced by adjusting the binding energy of adventitious C (1 s) to 284.62 eV. The Shirley-type background was subtracted before deconvolution and data fitting using the parameters used by Nesbitt et al [39]. For the multiplet peaks of Mn (2p_3/2_) for spectral fitting, a 20:80 ratio of the Lorentzian–Gaussian mix-sum function was used for all data fittings [35]. The sample morphology and nanostructure were determined by scanning electron microscopy (SEM) equipped with an energy-dispersive spectroscopy (EDS) detector (JSM-6390LV, JEOL, Japan) and high-resolution transmission electron microscopy (HRTEM, JEM-2100F, JEOL, Japan). The surface functional groups were analyzed by a Fourier transform infrared spectrometer (FT-IR, VERTEX 70, Bruker, Germany). Thermogravimetric analysis (TGA, NETZSCH TG 209 thermal analysis system, NETZSCH, Germany) was evaluated in a N_2_ atmosphere from room temperature (RT) to 800°C at a heating rate of ~10°C min^–1^. A Raman spectrum was obtained on a Renishaw InVia Raman spectrometer (Renishaw, Germany) under a backscattering geometry (the spectra were taken using the Ar^+^ lines, λ = 514 nm, the laser power in front of the microscope was 1 mW). Nitrogen adsorption−desorption was determined by Brunauer−Emmett−Teller (BET) tests using a Quantachrome Autosorb-1, JEDL-6390/LV (JEOL, Japan). The concentration of Mn in the material was determined by atomic absorption spectroscopy (AAS) using a flame atomic absorption spectrometer (HITACHI 180–80 HITACHI, Japan). Samples were digested overnight by NH_2_OH·HCl before testing.

### 2.4. Assays of the electrochemical properties

The material electrochemical performance was assayed using 2016 coin-type cells with lithium as the counter and reference electrodes at RT. The working electrode was fabricated by mixing the active material, acetylene black and polyvinylidene fluoride binder in a weight ratio of 8:1:1. The loading mass of each cathode is about 3 mg cm^−2^). The above mixtures were then pasted onto Cu foil (1.1 cm^2^). Each 1 mol L^–1^ LiPF_6_ in the ethylene carbonate−dimethyl carbonate mixture (1:1 by volume) was used as the electrolyte of coin cells, which were assembled in an argon-filled glove box with a microporous membrane (Celgard 2300) as a separator. The charge/discharge tests were performed on a battery tester (CT2001A Land Battery Testing System, Land, China) with galvanostatic in the voltage range of 0.01 to 3.0 V versus Li/Li^+^ at room temperature. Cyclic voltammogram (CV) tests of the electrode were carried out by using a CHI 660 c electrochemical workstation (Chenhua, Shanghai, China) with a scan rate of 0.1 mV s^–1^ between 0.01 and 3.0 V.

## 3. Results and discussion

### 3.1. Formation of BMC aggregates and characterization

**Figure 1.**
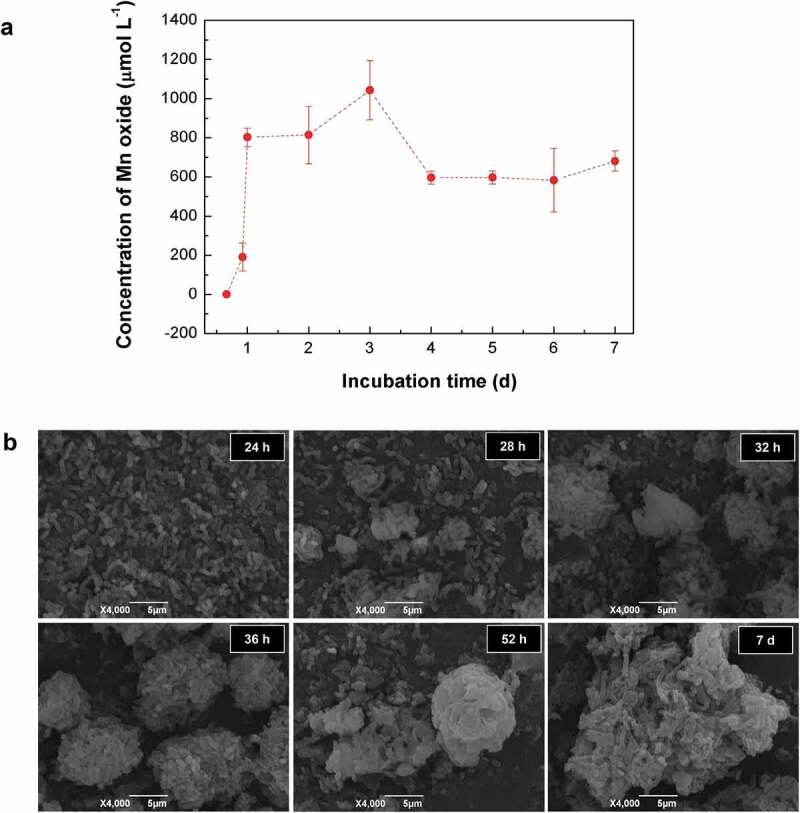
Kinetic analysis of (a) Mn^2+^ oxidation and (b) aggregate formation by wild-type strain T34

Kinetic monitoring of the whole-cell Mn^2+^-oxidizing activity during the culture of T34 showed an increasing Mn^2+^-oxidizing activity over the first 3 days, and it reached a maximum (1044 μmol L^–1^ of the equivalent MnO_2_) at 3 days. Then, it decreased to a relatively stable level (approximately 600–700 μmol L^–1^ of the equivalent MnO_2_) from 4 days to 7 days (Figure 1(a)). SEM observations indicated that T34 cells gathered gradually into aggregations after 24 h and then formed loose aggregates during the first 2 days. While the Mn(II) oxidation process occurs on the cell surface [5], the crystal growth of Mn biooxide by particle-attachment processes caused more compact microspherical aggregates on day 7 (Figure 1(b)) [40]. The 48 h aggregates were mainly composed of irregular flaky particles by stacking under SEM (Fig. S1a), with an irregular particle size from 8 μm to 15 μm. These aggregates (termed BMC) were porous, and the bacteria attached to or embedded in the aggregates were easily distinguished (Fig. S1b). The BET specific area and porosity analysis (Fig. S1c) showed that the specific surface area of the BMC was as high as 45.37 m^2^ g^–1^. The pore-size distribution curve based on the Barrett–Joyner–Halenda (BJH) model indicates that the major pore sizes included 15.6 nm, 23 nm and 34 nm, verifying that the prepared BMC is a nanoscale mesoporous material (with a normal pore size range of 0–50 nm) and the adsorption–desorption isotherm loop was of the H3-type (the inset in Fig. S1c). The EDS assay (Fig. S1d) shows a high Mn content (the mass fraction of Mn was 35.66%, Table S1) and the main organic matter nature of BMC. This agrees well with TGA analysis (Fig. S2a), which validated that ~9% of attached water was lost below 100°C and 53.24% of the organic matter from bacteria was decomposed when the temperature varied from 100°C to 800°C. Trace elements of Ca, Na and Zn can also be detected, however, the contents are extremely low, which can almost be ignored. Hereby, the trace elements of Na and Zn from bacteria have no effect on the phase composition of the carbonized material. The Cu element comes from the copper TEM grid. The accurate concentration of Mn in BMC was 36.25 wt%, which was further evaluated by atomic absorption spectroscopy (Fig. S3) and was also consistent with the above results.

**Figure 2.**
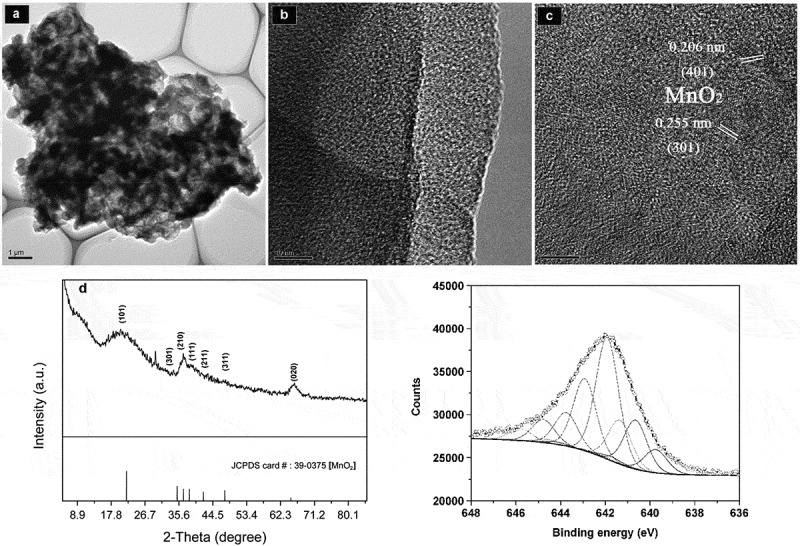
(a) TEM images of BMC. (b) High-magnification TEM image taken from the edge of an individual BMC. (c) Typical HRTEM image of BMC and measured crystal lattice spacings. (d) XRD patterns of BMC. (e) XPS patterns of the Mn(2p_3/2_) spectrum of Mn oxides in BMC. The upper circles represent observed data. The thick, solid curve indicates the best fit of the data. The dashed–dotted curves represent Mn^4+^ multiplet peaks, the dotted lines represent Mn^3+^, and the thin solid lines represent Mn^2+^

A single aggregate particle, as observed by HRTEM, was formed by the mutual stacking of rod bacteria and flaky Mn oxides, and the edges were wrapped by organic carbon layers (Figure 2(a,b)). Mn-oxide nanoparticles exhibited a good dispersity, as verified by many groups of crystal particles of 5 ± 1 nm that were embedded independently in the carbon matrix. The lattice fringes of 0.206 nm and 0.255 nm corresponded to a *d* value of (401) and (301) plane spacings in ramsdellite-type MnO_2_, respectively. The XRD spectra of BMC showed that the diffraction peaks were indexed to the (101), (301), (210), (111), (211), (311) and (020) planes of ramsdellite-type MnO_2_, respectively (Figure 2(c,d)). The relative contents of Mn^2+^, Mn^3+^ and Mn^4+^ on the Mn-oxide surface in the BMC were determined by XPS. The Mn(2p_3/2_) spectra of the Mn oxides in BMC is shown in Figure 2(e). The spectral fittings of the near-surface composition indicate that the Mn oxides contained Mn^2+^, Mn^3+^ and Mn^4+^, with percentages of 9.48%, 24.47% and 66.05%, respectively (Table S2). FT-IR analysis (Fig. S4) indicated that the Mn-O bond, corresponding to the vibration peak at 515 cm^–1^ [41] was not detected for either T34 cells alone or in the initial suspension of cell-MnCl_2_ solution, but it clearly appeared for the BMC. These results verify that low-valence Mn was oxidized to high-valence Mn after T34 mineralization, with the major type of Mn oxide being MnO_2_.

### 3.2. Preparation and Characterization of MnO/C/Co_3_O_4_ Composites

**Figure 3.**
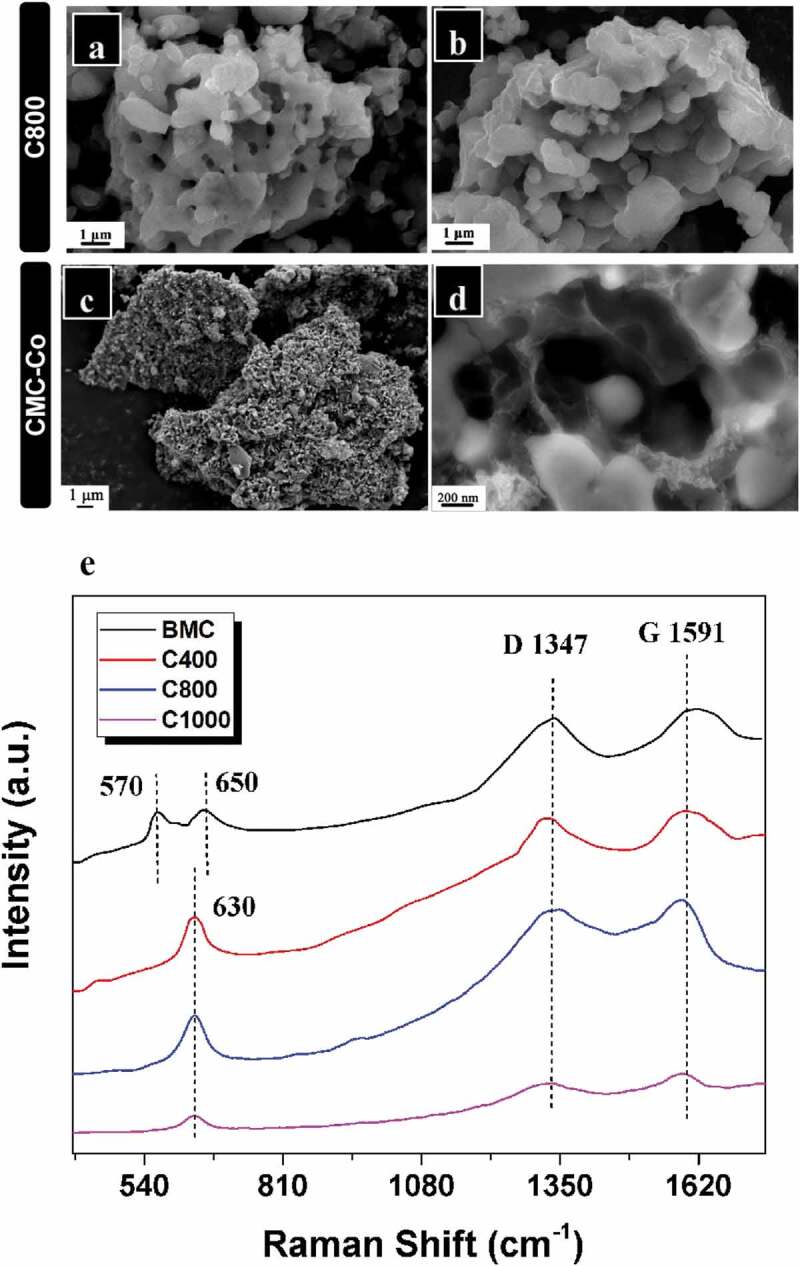
SEM images of (a, b) material prepared at 800°C (C800) and (c, d) composite material (CMC-Co). (e) Overview of the Raman spectra of BMC, C400, C800 and C1000

The Co^2+^ ions were added into a T34 suspension incubated for 24 h with enrichment by 1 mmol L^–1^ MnCl_2_. By biological ion exchange and a redox reaction of Mn oxidation, cation-incorporated biogenic Mn oxide aggregates were obtained when the mixed suspensions were incubated for a further 24 h. Biogenic Mn oxides were then transferred into a carbonization furnace for calcination and annealing. SEM shows typical morphologies of the resultant materials using BMC as the precursor after thermal treatment in Ar at 800°C. The particle sizes at carbonization temperatures of 400°C (C400) and 800°C (C800) were approximately 7–10 μm, but when the temperature was increased to 1000°C (C1000), the particle size decreased remarkably to only 4–8 μm (Figure 3(a), Fig. S5a,c). Although the particle size appeared to be reduced after high-temperature calcination, the overall structure of the precursors exhibited no significant change, and the particles remained approximately spherical, which indicates that the aggregate structure formed by self-assembly after T34 Mn oxidation was in a stable state. The porous structures were visualized easily from their surfaces; however, specific particles with a hollow structure were formed only at 800°C (Figure 3(b), Fig. S5b). It is worth noting that organic matter can not be completely removed at 400°C (Fig. S2b), however, the organic matter content in the C800 material can decreased significantly to ~7% (Fig. S6). These results suggest that the hollow porous structure, which was formed by gas release due to the decomposition of organic matter at high temperature, could be obtained only at a suitable carbonization temperature (800°C), whereas a higher annealing temperature (1000°C) may result in the collapse of the internal material skeleton (Fig. S5D). Compared with the materials derived from BMC, the particle volume of composites derived from BMC-Co exhibited no significant change. The hollow porous structures remained, but their surfaces were rougher with many small-particle attachments (Figure 3(c,d)). We speculate that these attached particles were mainly composed of Mn and Co after reaction at high temperature. The EDS results also verified that the main elements in the CMC-Co included Mn, O, C, Co, P and trace Al (Fig. S7). The production yield of C800 and CMC-Co nanocomposites under the annealing temperature of 800°C was 37.76%, which was derived from the TGA results of BMC (Fig. S2).

Raman spectrum analysis was performed to examine the influence of the carbonization temperature on the carbon-matrix graphitization of the composites (Figure 3(e)), and two peaks which are characteristic peaks of the Mn-O lattice were observed at 570 and 650 cm^−1^ for BMC [42], while the vibration mode was visible at 630 cm^–1^ for C400, C800 and C1000. The characteristic peaks of Mn oxide after carbonized (C400, C800 and C1000) belongs to Mn_3_O_4_ rather than MnO_2_ or MnO [34]. Since the samples are sensitive to the laser and it may cause laser oxidation, the Raman characteristic peak of MnO will easily transform to Mn_3_O_4_ because of the local heating effect and photochemically induced transformations under beam irradiation, which explains the reason for the lack of Mn-O peaks from MnO_2_ and MnO [43,44]. In the high-frequency region, two obvious Raman peaks at 1347.02 cm^–1^ or 1591.18 cm^–1^ were visible for C800, and they were also distinguished for BMC, C400 and C1000, which corresponded to the A_1g_ vibration mode (D bond) of amorphous carbon and the E_2g_ vibration mode (G bond) of orderly graphite carbon, respectively. The I_D_/I_G_ strength ratios were 0.981, 0.975, 0.958 and 0.905 for BMC, C400, C800 and C1000, respectively. Therefore, these materials contained graphitized carbon (SP^2^) and amorphous carbon (SP^3^) hybrid carbon atoms. According to the SEM and Raman spectral analysis results, the materials obtained at a carbonization temperature of 800°C (C800 and CMC-Co) exhibited better morphology and contained graphitized carbon, and therefore, they were selected for subsequent studies.

**Figure 4.**
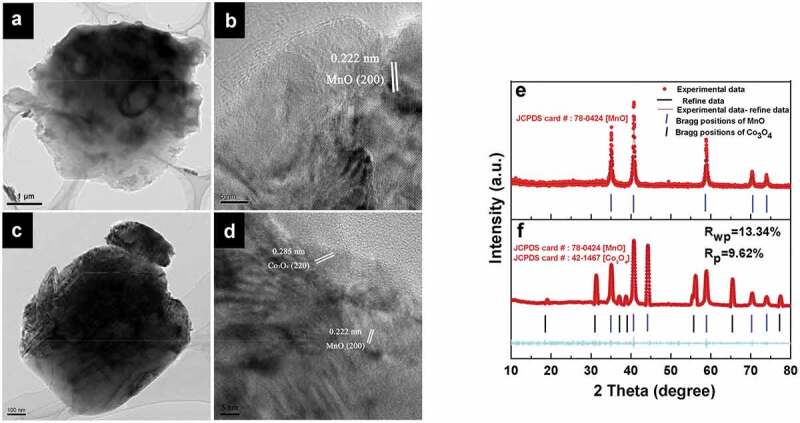
HRTEM and corresponding XRD patterns of materials prepared at 800°C. In the HRTEM micrographs, (a) and (c): the structures of C800 and CMC-Co; (b) and (d): the measured crystal lattice spacings of C800 and CMC-Co. (e) and (f): XRD patterns of C800 and CMC-Co. Red dots, black lines, magenta lines and blue bars represent experimental and refined data, their difference and the Bragg positions, respectively

The microstructure of the materials was also analyzed by HRTEM. The spherical structures of materials were unchanged by the incorporation of additional elements at a carbonization temperature of 800°C (Figure 4(a,c)). The image taken from the outside edge of the C800 clearly demonstrates that the unique porous structure is composed of MnO nanocrystals anchored into the amorphous carbon matrix with a distinct interplanar spacing of 0.222 nm, which is attributed to the (200) planes of MnO (Figure 4(b)). In addition to the lattice fringe of MnO, special lattice fringes of Co_3_O_4_ in the composite material were visible. The interplanar spacing of 0.285 nm in CMC-Co was attributed to the (220) planes of Co_3_O_4_ (Figure 4(d)). New generated phases in CMC-Co were identified by Rietveld refinement analyses to investigate the phase transformation of materials after carbonization. As shown in Figure 4(e), during annealing at 800°C, the reducing atmosphere because of biological carbon decomposition rendered a BMC phase change. MnO_2_ in the BMC precursor was converted to MnO. The diffraction peak of MnO was clear and sharp, which indicates the good formation of crystals. Refinement data of CMC-Co show that MnO and Co_3_O_4_ were the major phases, which corresponded to MnO [JCPDS 78–0424, space group Fm-3 m (225), a = 4.445 Å] and Co_3_O_4_ [JCPDS 42–1467, Fd-3 m (227), a = 8.056 Å]. In order to further confirm the above result, we further performed a multi-phase Rietveld refinement. The model used for the refinement was based on the Co_3_O_4_ components. The procedure involved varying Mn occupancy and Co/O ratio, respectively. A good fit was obtained and the Rietveld analysis indices were Rwp = 13.34% and Rp = 9.62% (Figure 4(f)). The lattice parameter of MnO in CMC-Co was the same as that of the C800, *i.e*. 4.445 Å. However, second-phase Co_3_O_4_ was formed. The average crystal sizes of MnO in C800 and CMC-Co are 26 nm and 32 nm, respectively, while the average size of Co_3_O_4_ in CMC-Co was 31.5 nm, as estimated from the Scherrer equation. The phase concentrations for MnO and Co_3_O_4_ were 88.5% and 11.5%, respectively, as calculated from the XRD pattern. The presence of multiple phases was in good agreement with the HRTEM data. The changes in the Mn species of the materials were also analyzed by XPS. The Mn2p_3/2_ XPS fitting data indicate that C800 contained three valence states of Mn, *i.e*. Mn^2+^, Mn^3+^ and Mn^4+^ (Fig. S8a). According to the peak intensities, the corresponding proportions of Mn^2+^, Mn^3+^ and Mn^4+^ were calculated as 89.09%, 5.51% and 5.40%, respectively (Table S2), which indicates that the main oxidized state of the Mn ions was converted from Mn^4+^ to Mn^2+^ after high-temperature reduction. In addition to changes in the Mn species, the XPS 2p_3/2_ fitting data of CMC-Co also verified the presence of Co_3_O_4_ (Fig. S9). These results were consistent with the XRD analysis. The parameters used for fitting referred to references, and the corresponding proportions are listed in Table S3 [45–47]. The contents of Mn^3+^ and Mn^4+^ increased slightly in CMC-Co after annealing (Table S2, Fig. S8b), probably because the partial reduction potentials were shared by the Co reactions during the annealing processes.

### 3.3. Electrochemical determination of composites as anodes for lithium-ion batteries

**Figure 5.**
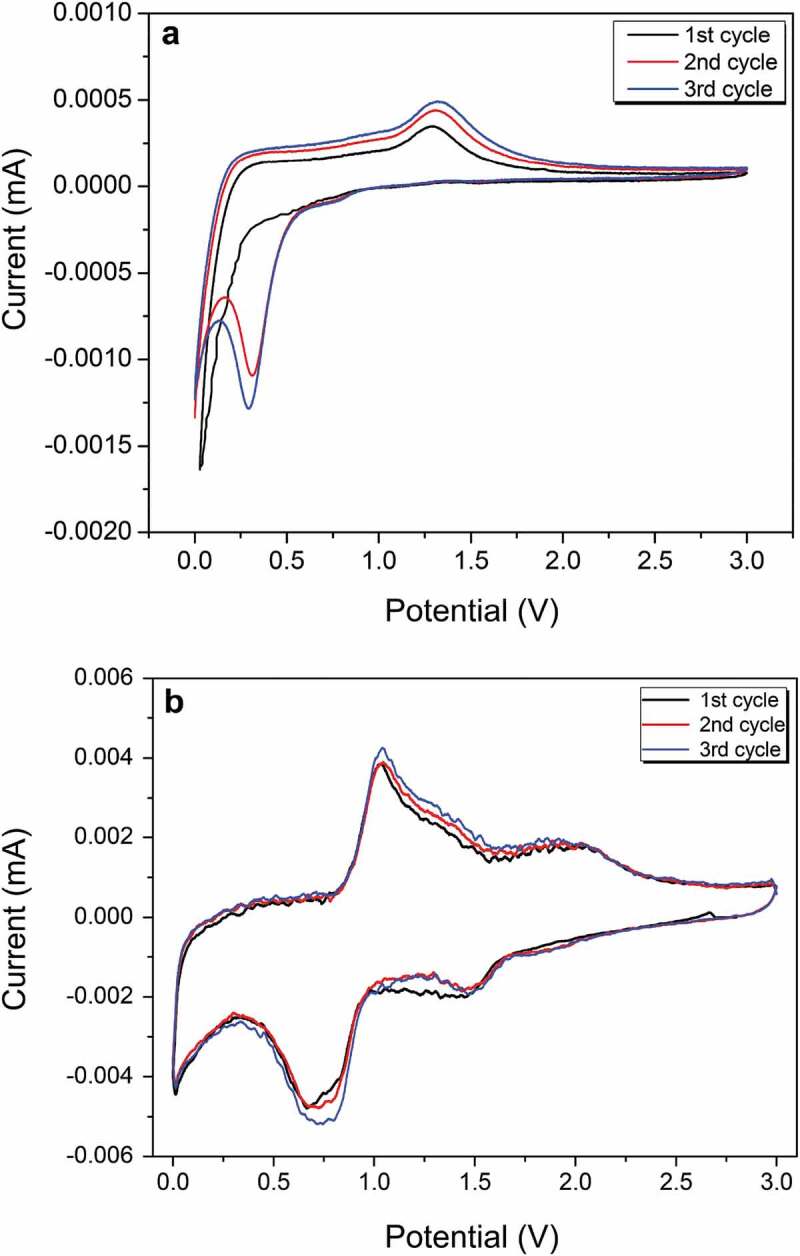
CV curves of (a) C800 and (b) CMC-Co at 0.1 mV s^–1^

The cycling performance and rate capacity of anode materials using the prepared composites for lithium-ion batteries were investigated. The active-material mass was defined as the total mass of amorphous carbon and metal oxides to normalize the determination and calculation of the specific capacity. The electrochemical reaction of the prepared materials under 800°C carbonization was analyzed using cyclic voltammetry. Figure 5 shows that the sharp reduction peak close to 0.1 V in the first cathodic sweep agrees well with the reduction of Mn^2+^ to Mn^0^ and the formation of solid electrolyte interface (SEI) layers for the C800 material without Co_3_O_4_ [48]. After the second cycle, the reduction peak shifted to the left to 0.3 V, which indicates an irreversible phase transition of Li_2_O and metallic Mn [34]. The anode peak at 1.25 V during anode scanning occurred because of Mn^0^ oxidization to Mn^2+^ and Li_2_O decomposition. Compared with C800, the first reduction peak shifted to the right to 0.7 V for the CMC-Co, which may have been caused by a multiphasic composite that formed by Co_3_O_4_ and MnO. This reduction peak was likely from the formation of a SEI layer and two reduction reactions, i.e. from Mn^2+^ to Mn^0^ and CoO to Co [49]. The second reduction peak at 1.5 V presented oxidization reactions from Mn^0^ to Mn^2+^ and from Co^0^ to Co^2+^, whereas the anode peak at 2.1 V resulted because of CoO oxidization to Co_3_O_4_ [49] (Figure 5).

**Figure 6.**
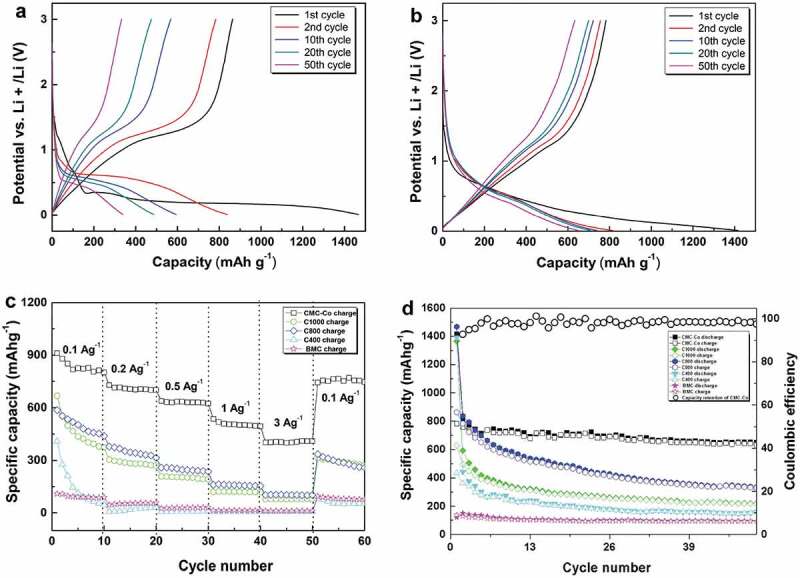
(a,b) Charge–discharge profiles of C800 and CMC-Co at different cycles with a current density of 0.1 A g^–1^. Comparison of the (c) rate performance of CMC-Co, C1000, C800, C400, and BMC electrodes at different current densities and (d) their cycling performance at a current density of 0.1 A g^–1^. All were tested from 0 V to 3.0 V (*vs*. Li/Li^+^)

The constant current (0–3 V, *vs*. Li/Li^+^) charge–discharge profiles of the prepared materials with a carbonization temperature of 800°C at a current density of 0.1 A g^–1^ are shown in Figure 6. The coulombic efficiency of the first cycle was 58.8% for C800, which indicates a considerable irreversible capacity loss of this cycle. The formation of the solid electrolyte interphase (SEI) film, incomplete disengagement of Li, and structural degradation are the causes of capacity fading [50]. A long and smooth discharge plateau was observed near 0.25 V in the first discharge process, which was attributable to the formation of a SEI layer and Mn^2+^ reduction to Mn^0^. However, the discharge plateau shifted to ~0.5 V from the second cycle, which indicates the polarization phenomenon of C800 during the discharge process. A charge plateau occurred at 1–1.5 V during delithiation, which corresponded to the oxidization reaction from Mn^0^ to Mn^2+^ (Figure 6(a)). The discharge–voltage plateau for CMC-Co was at 0.7 V (Figure 6(b)). The charge–voltage plateau existed near 1.3 V. The charge plateau and discharge plateau correspond to the phase-change reaction of MnO, which was the major material component. The charge–discharge curves with different cycle times show that the change in the voltage plateau was small for the composite materials even after 50 cycles since the second cycle and reflects that the voltage-delay effect disappeared by introducing Co_3_O_4_. The low charge plateau at 1.3 V indicated that a high open-circuit voltage and energy density could be obtained after assembly with specific anodes. The first discharge capacity for CMC-Co electrodes was 1416.4 mAh g^–1^. The coulombic efficiency in the first cycle was 55.3%. Therefore, the first irreversible loss was still considerable. At a current density of 0.1 A g^–1^, the remaining reversible discharge capacity for CMC-Co after 50 cycles was 650 mAh g^–1^, which was significantly improved compared with the 335.2 mAh g^–1^ of C800. These results demonstrate that the CMC-Co exhibits a significantly improved electrochemical property over C800 in terms of a better cycle stability and energy efficiency, which occurs mainly because of the formation of the multiphasic MnO-based composite.

C800 exhibited a better cycling performance and better rate performance compared to C1000, C400 and BMC (Figure 6(c)). When the current density was 0.1, 0.2, 0.5, 1, 3 and 0.1 Ag^–1^, the reversible charge specific capacity values of C800 were 502.7, 346, 246.7, 159.4, 103.8 and 289.9 mAh g^–1^, respectively. However, a rapid capacity fade was still observed for the C800 sample. Under similar rate-testing conditions, the performance of CMC-Co was improved significantly, and it showed the best rate performance, for which the reversible specific capacities were 825.2, 706.4, 634.2, 505.2, 400.8 and 765.6 mAh g^–1^, respectively. Figure 6(d) shows the specific capacity changes of samples within 50 cycles at a current density of 0.1 A g^–1^. As a control, the initial discharge capacity of noncarbonized BMC was 120.03 mAh g^–1^, whereas the reversible discharge capacity after 50 cycles was only 94.16 mAh g^–1^. It is likely that the presence of massive functional groups and cation vacancies in BMC led to a very low internal electronic conductivity (Fig. S4) [19,49]. Moreover, the bound water components within the organic matter could cause a substantial increase in the contact resistance and decomposition of electrolyte lithium salt, which further deteriorated the cycling performance of the battery [51]. In contrast, the cycling capacity of all high temperature-carbonized composites increased significantly. The initial discharge capacities of C1000, C800 and C400 were 1364.6 mAh g^–1^, 1468.6 mAh g^–1^ and 1390.8 mAh g^–1^, respectively. The improvement in the initial discharge capacity could result from an enhancement in carbonaceous graphitization after high-temperature annealing, a decrease in cation vacancies and the removal of bound water components. The additional discharge capacity compared with the theoretical capacity of MnO (756 mAh g^–1^) could result from the decomposition of the electrolyte during the formation of a solid electrolyte interface (SEI) layer and graphite carbon [52]. The direct embedding of MnO nanocrystals in conductive porous carbon could form firm connections, enhance the electrical contact between MnO and carbon, and reduce the reaction resistance [23,34]. However, the results also show that the discharge capacities of all of the prepared materials were subjected to fast attenuation during the cycle process, even for C800 with the highest initial discharge capacity. When the current density was 0.1 A g^–1^, the reversible specific capacity of C800 remained at only 335.2 mAh g^–1^ after 50 cycles. The capacity degradation in the cyclic process normally occurs in Mn-oxide materials used in lithium batteries because of the irreversible phase transition caused by the Jahn–Teller effect and the disproportionation reaction that is caused by Mn dissolution into the electrolyte [53]. In contrast, the CMC-Co electrode exhibited distinguished cycle stability from the second cycle. CMC-Co maintains a high coulombic efficiency that is stabilized at approximately 99% along with an increase in the cycle number (Figure 6(d)).

Undoubtedly, a high maximum specific discharge capacity towards lithium-ion batteries is an appealing feature of these materials. Several previously described MnO-based systems exhibited higher electrochemical capacities, with maximum specific capacities of 500 mAh g^–1^ (0.189 Ag^–1^, 25 cycles) for the coaxial MnO/C nanotubes, 610 mAh g^–1^ (0.2 Ag^–1^, 60 cycles) for a MnO/C microtube material [30] and 651.8 mAh g^–1^ (0.1 A g^–1^, 90 cycles) for a carbon-coated Fe-Mn-O composite [24]. In comparison, the maximum reversible discharge capacity of the CMC-Co prepared in this study reached 650 mAh g^–1^ (0.1 A g^–1^, 50 cycles), which is a comparable capacity to the available MnO-based materials with higher levels (Table S4). The better electrochemical properties of these prepared materials mainly result because of the optimized carbon network structure. The hollow porous structure provided a large reaction area between the active materials and electrolyte and shortened the diffusion distance of the lithium ions. A suitable carbonization temperature improved the conductive properties of the material. The mutltiphase coexistence of several oxides restricted the aggregation and dissimilation phenomenon of materials in electrochemical lithiation and deintercalation cycles. Therefore, the stability and capacity in cycle processes were further improved. It is also worth noting that the easy and cost-effective preparation of these materials indicates their potential use in the bioenergy field.

## 4. Conclusions

A facile fabrication method of hollow porous MnO/C/Co_3_O_4_ nanocomposites by a biotemplated strategy was proposed for the first time. The electrochemical properties of the prepared biocomposites as anode materials for lithium-ion batteries were enhanced by a unique biological incorporation route. The biogenic Mn-oxide aggregate structure formed from *Pseudomonas* sp. T34 with a high Mn^2+^-oxidizing activity was used as the precursor. The incorporation of Co under mild conditions was realized through the biomineralization of biogenic Mn oxides and the characteristics of the metal-ion settler. Hollow porous MnO/C/Co_3_O_4_ multiphasic composites were fabricated by decomposing strains through high-temperature annealing. The composites exhibit an excellent cycling performance and energy efficiency in electrochemical testing, the reversible discharge capacity of CMC-Co remained at 650 mAh g^–1^ after 50 cycles at a current density of 0.1 Ag^–1^, and the coulombic efficiency remained above 99% after the second cycle, which indicates their potential application in lithium-ion batteries. Our study explores the feasibility of biomineralization in the construction of electrode materials for lithium batteries. However, there are also problems in this method such as the long time it took to produce the Mn biooxide and the yield of CMC-Co from BMC-Co is not very high, but these could be improved by constructing an engineered strain with a higher Mn^2+^-oxidizing activity or optimizing the culture conditions in further experiments. The new biogenic materials and special biological incorporation route could be used in the bioremediation of heavy metals and in other fields.
